# Supplementary material for: Activating cobalt(II) oxide nanorods for efficient electrocatalysis by strain engineering
Source: Nat Commun. 2017 Nov 15;8:1509. doi: 10.1038/s41467-017-01872-y (PMC5686154; doi:10.1038/s41467-017-01872-y)
Supplement: Supplementary file 1 — Supplementary Information [file 41467_2017_1872_MOESM1_ESM.pdf]

Supplementary Figures

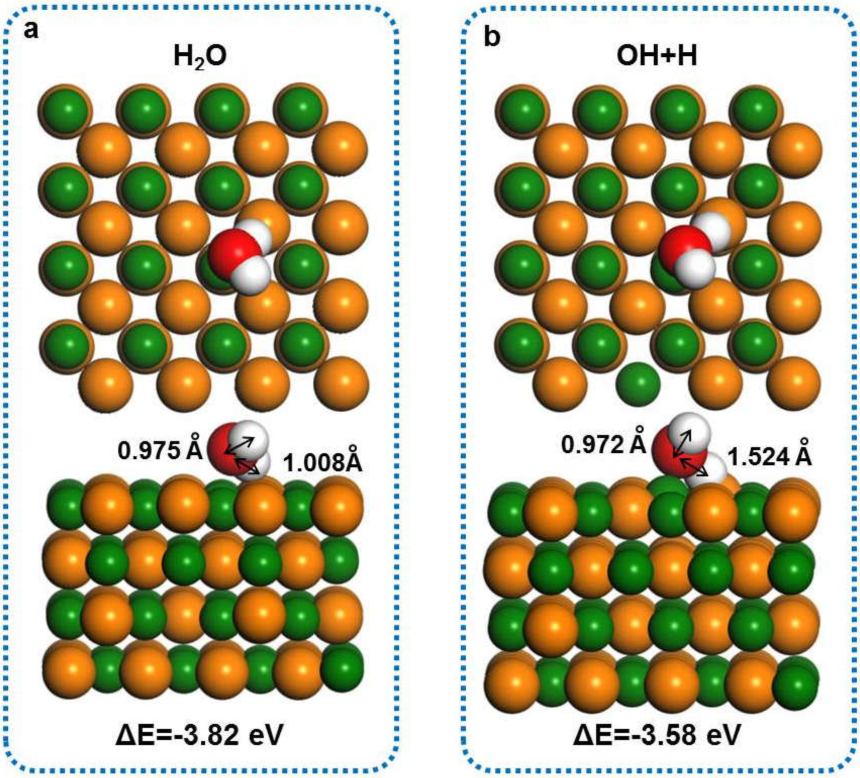

Supplementary Figure 1. Atomic configurations of H_2O (a) adsorbed and (b) dissociated on the CoO {100} surface. The corresponding adsorption energies are provided at the bottom of panels (a) and (b). Color codes: white and red spheres denote respectively hydrogen and oxygen atoms in water molecule, and orange and green spheres denote respectively oxygen and cobalt atoms in CoO.

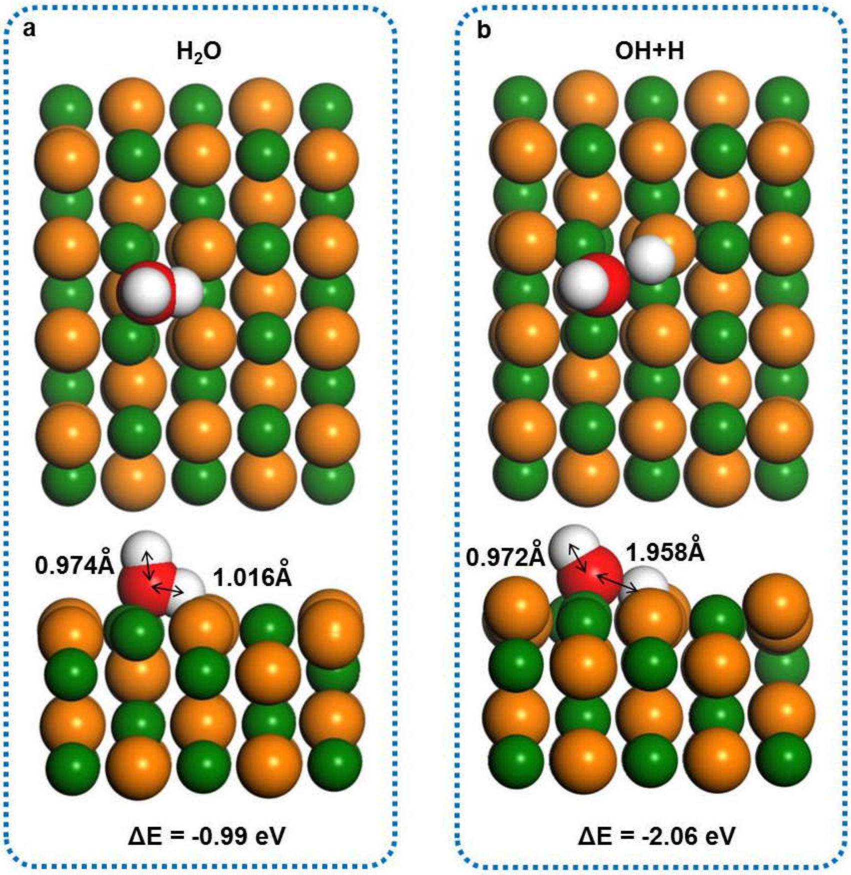

Supplementary Figure 2. Atomic configurations of H_2O (a) adsorbed and (b) dissociated on the CoO {110} surface. The corresponding adsorption energies are provided at the bottom of panels (a) and (b). Color codes are the same as in Supplementary Fig. 1.

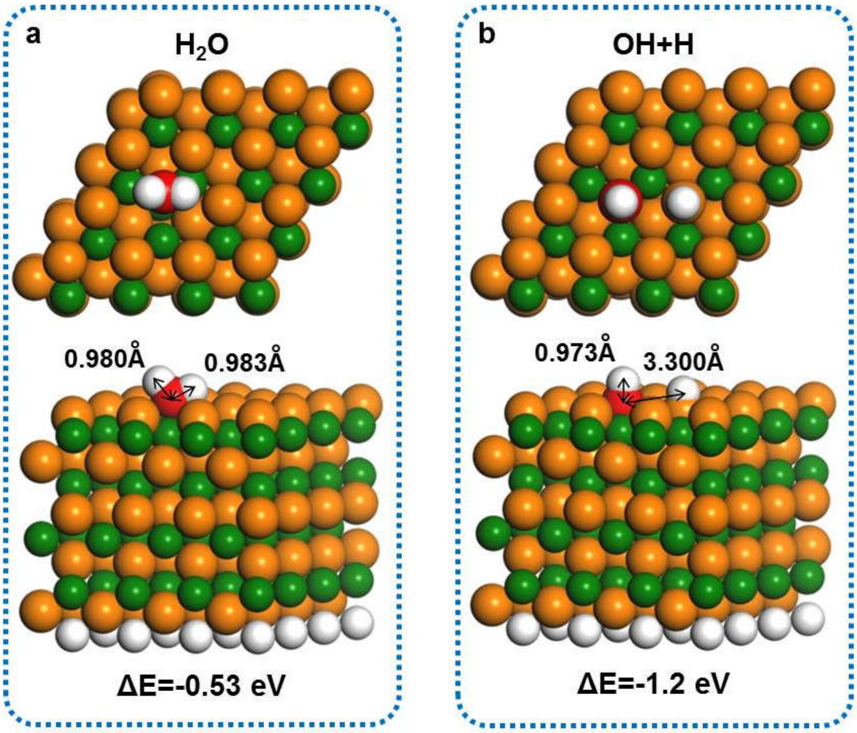

Supplementary Figure 3. Atomic configurations of H_2O (a) adsorbed and (b) dissociated on the CoO {111}-Ov surface. The corresponding adsorption energies are provided at the bottom of panels (a) and (b). Color codes are the same as in Supplementary Fig. 1.

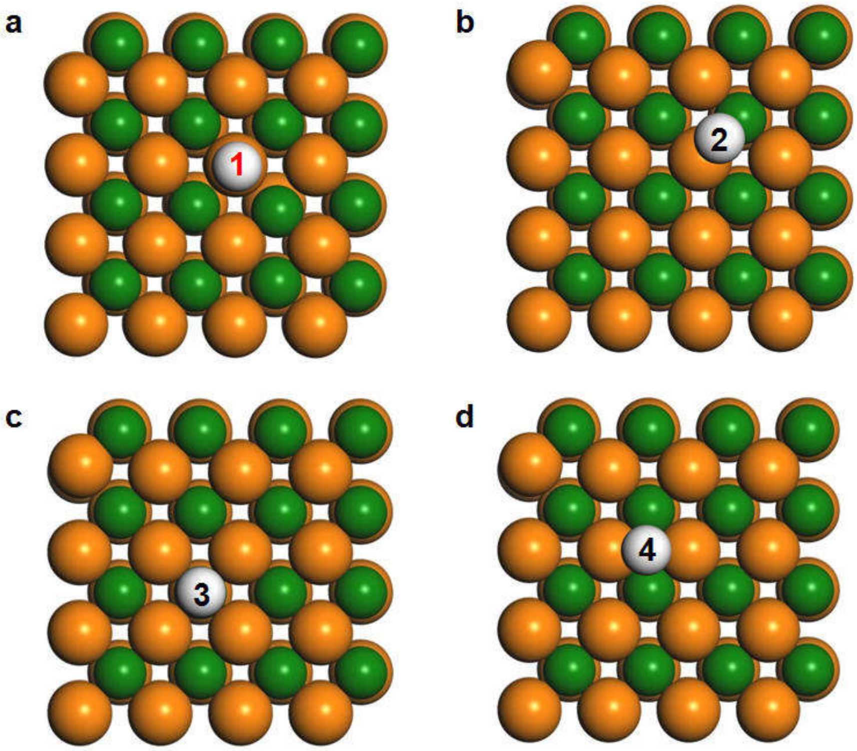

Supplementary Figure 4. Investigation of possible adsorption sites on the CoO {100} surface. a-d show the models of the aforementioned surface with 1-4 adsorption sites, respectively. The most stable adsorption site is 1 with the adsorption energy (E_d) of -2.81 eV. Color codes: orange and green denote oxygen and cobalt atom, respectively.

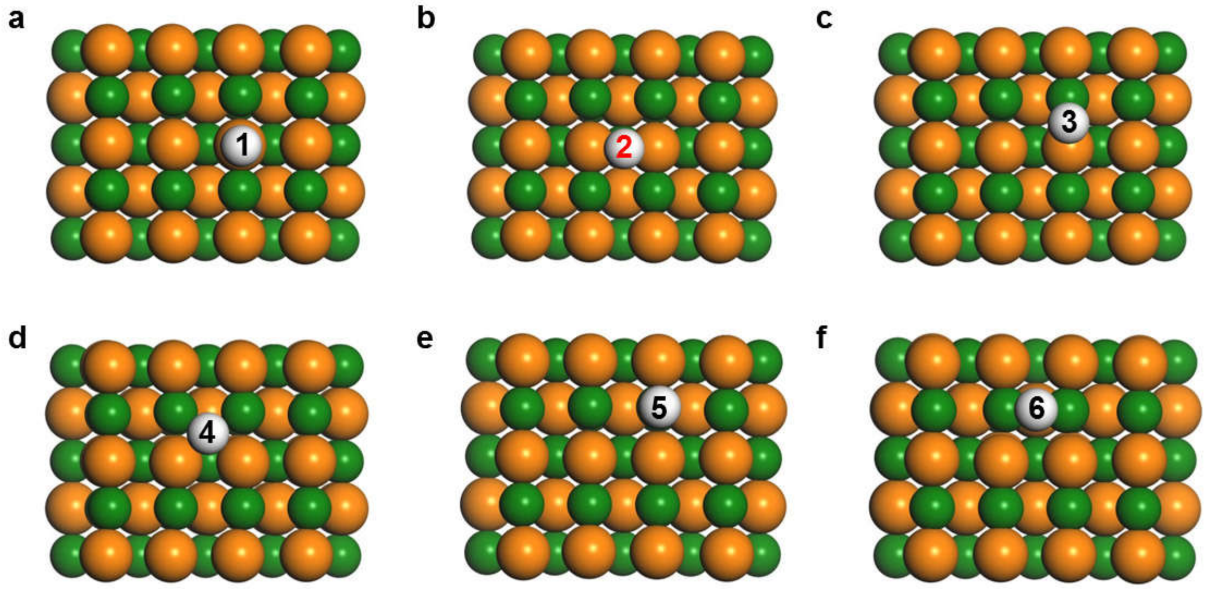

Supplementary Figure 5. Investigation of possible adsorption sites on the CoO {110} surface. a-f show the models of the aforementioned surface with 1-6 adsorption sites, respectively. The most stable adsorption site is 2 with E_d = -2.13 eV. Color codes are the same as in Supplementary Fig. 4.

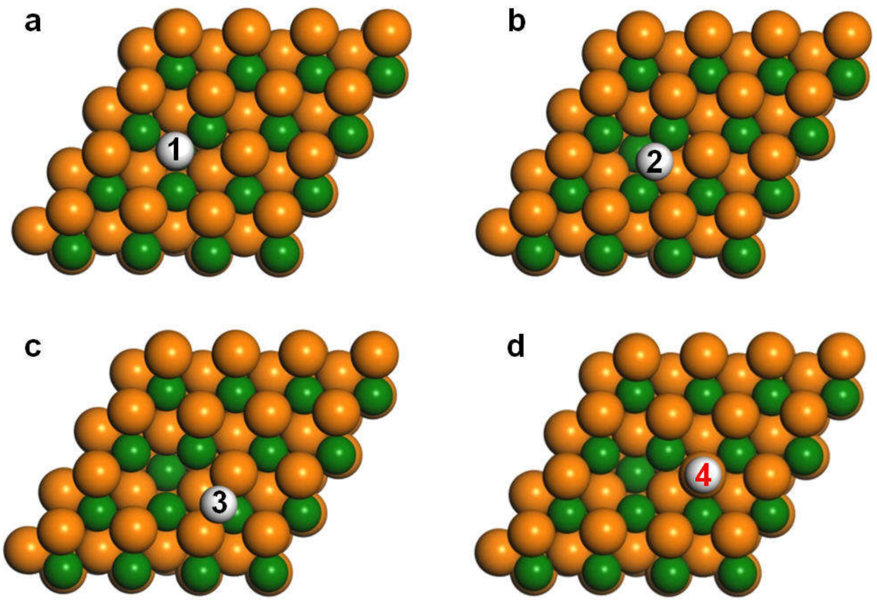

Supplementary Figure 6. Investigation of possible adsorption sites on the CoO {111}-Ov surface. a-d show the models of the aforementioned surface with 1-4 adsorption sites, respectively. The most stable adsorption site is 4 with E_d=-1.52 eV. Color codes are the same as in Supplementary Fig. 4.

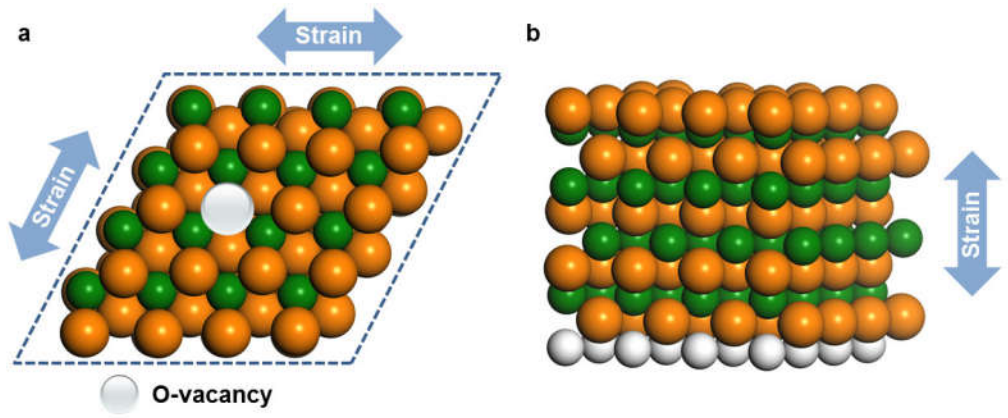

Supplementary Figure 7. Computational super cell of CoO {111}-Ov under tensile strain. a and **b**, Top view and side view of the atomic structures of {111}-Ov with the surface O-vacancy concentration of 11.1 %. The arrows indicate the directions of elastic strain. Color codes: orange and green spheres denote oxygen and cobalt atoms, respectively, and white spheres at the bottom of the super cell denote hydrogen layer, which was introduced to make the slab to obey the electron counting rule.

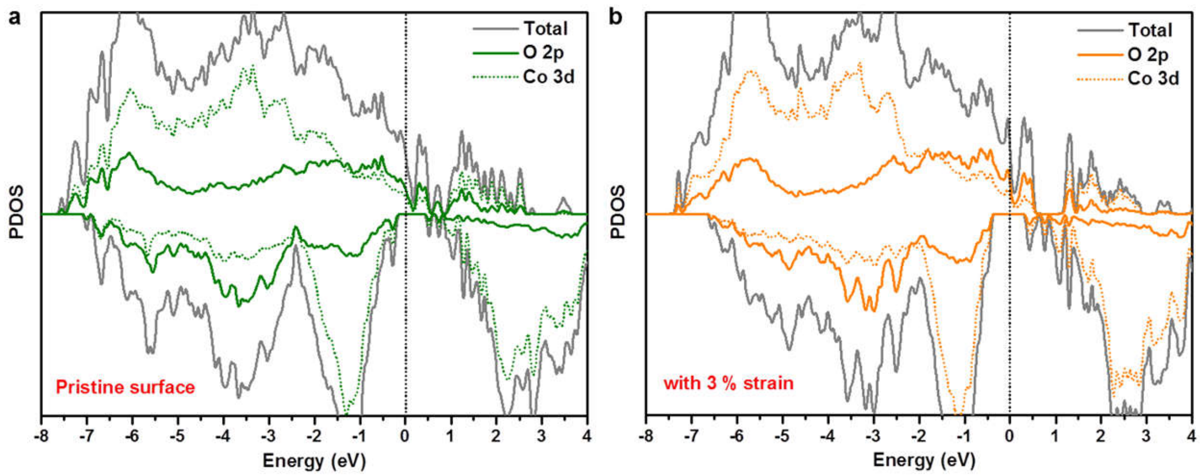

Supplementary Figure 8. Investigation of the electronic structure of pristine and 3.0 % strained CoO. a and **b**, Projected density of states (PDOS) for the pristine and strained surfaces, respectively.

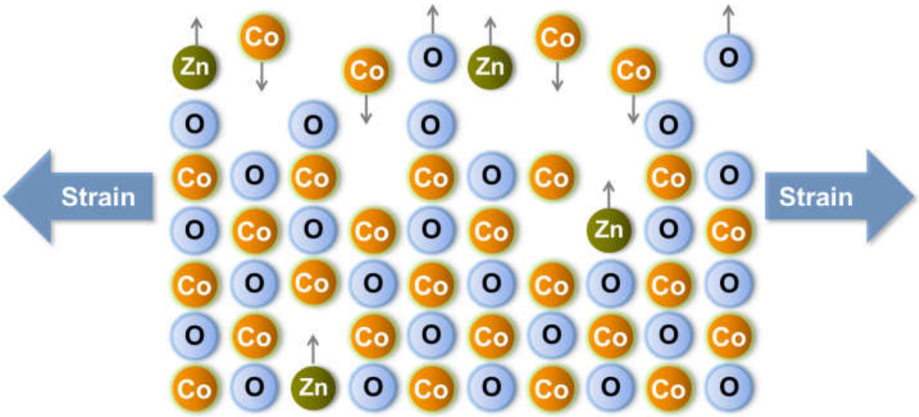

Supplementary Figure 9. Schematic illustration of creation of O-vacancies and strain on the surface of CoO (viewed from [100] direction) by cation exchange methodology. The presence of surface strain on CoO NRs facilitates the Co^{2+} inward diffusion and Zn^{2+} outward diffusion through O-vacancies, which results in the fast cation exchange[1,2]. Notably, at the initial stage of cation exchange, hexagonal ZnO and face-centered cubic (fcc) CoO coexist in the exchanged product. As replacement reaction of the Co^{2+} cations for Zn^{2+} cations proceeds, phase transition happens, and the residual Zn^{2+} ions are well-integrated into the fcc lattice of CoO to form solid solution $Zn_xCo_{1-x}O$.

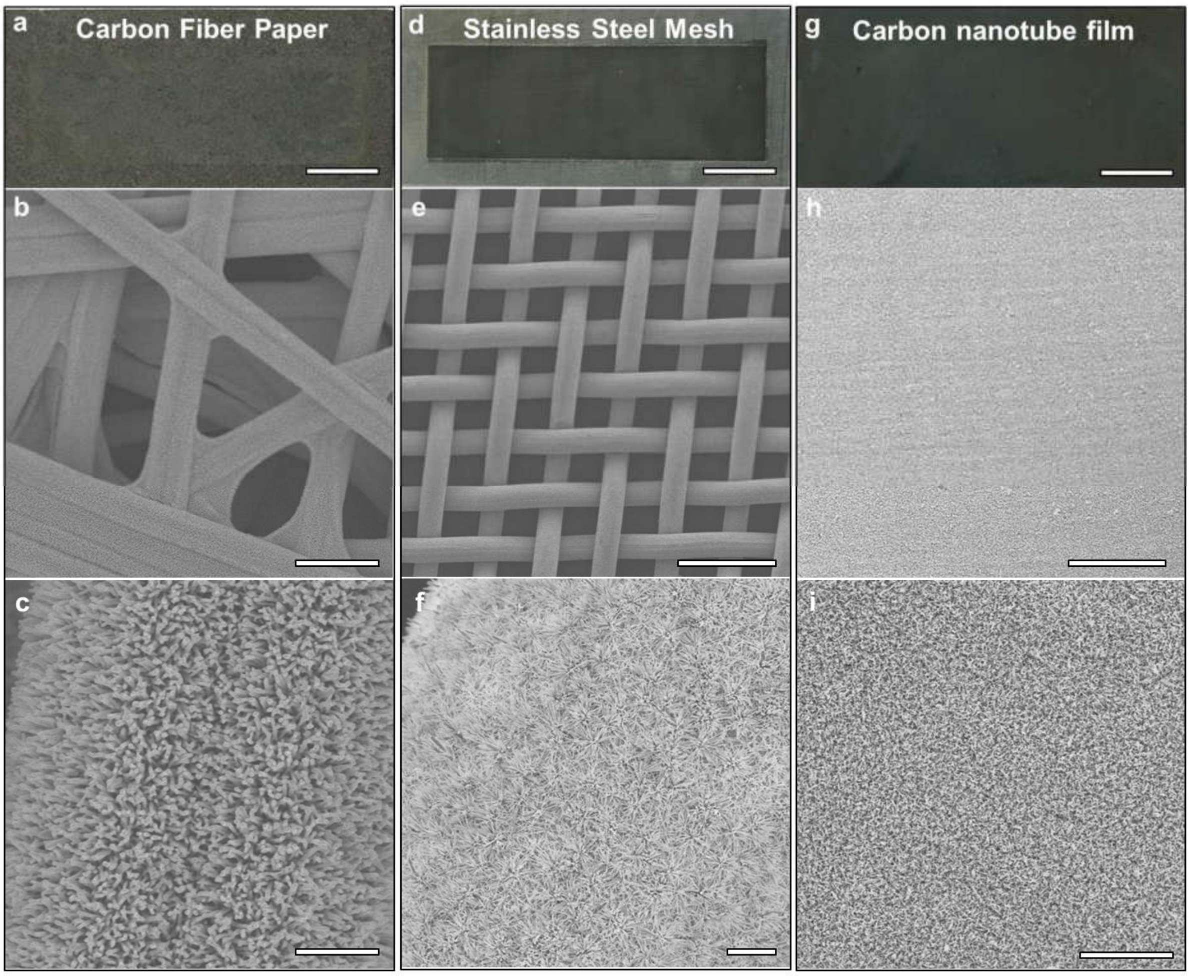

Supplementary Figure 10. Controllable fabrication of S-CoO NRs on various conductive substrates. a-c, **d-f**, and **g-i**, S-CoO NRs on carbon fiber paper (CFP), stainless steel mesh and carbon nanotube film, respectively. Scale bars in **a**, **d** and **g**, 1 cm. Scale bars in **b**, **e** and **h**, 20, 200 and 20 μm, respectively. Scale bars in **c**, **f** and **i**, 2, 4 and 4 μm, respectively.

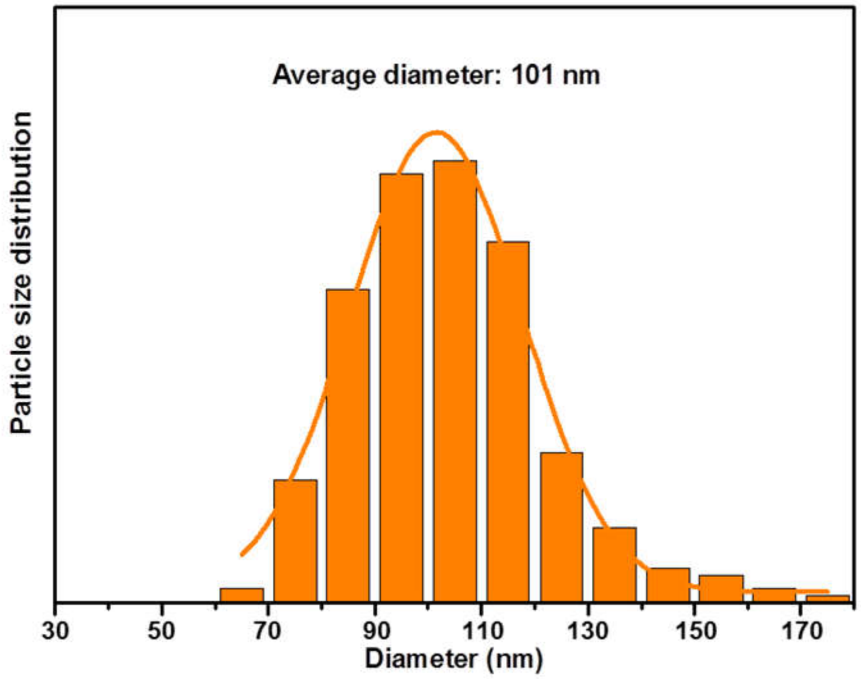

Supplementary Figure 11. Particle size distribution of S-CoO NRs fabricated directly on CFP using cation exchange at 600 ^{o}C.

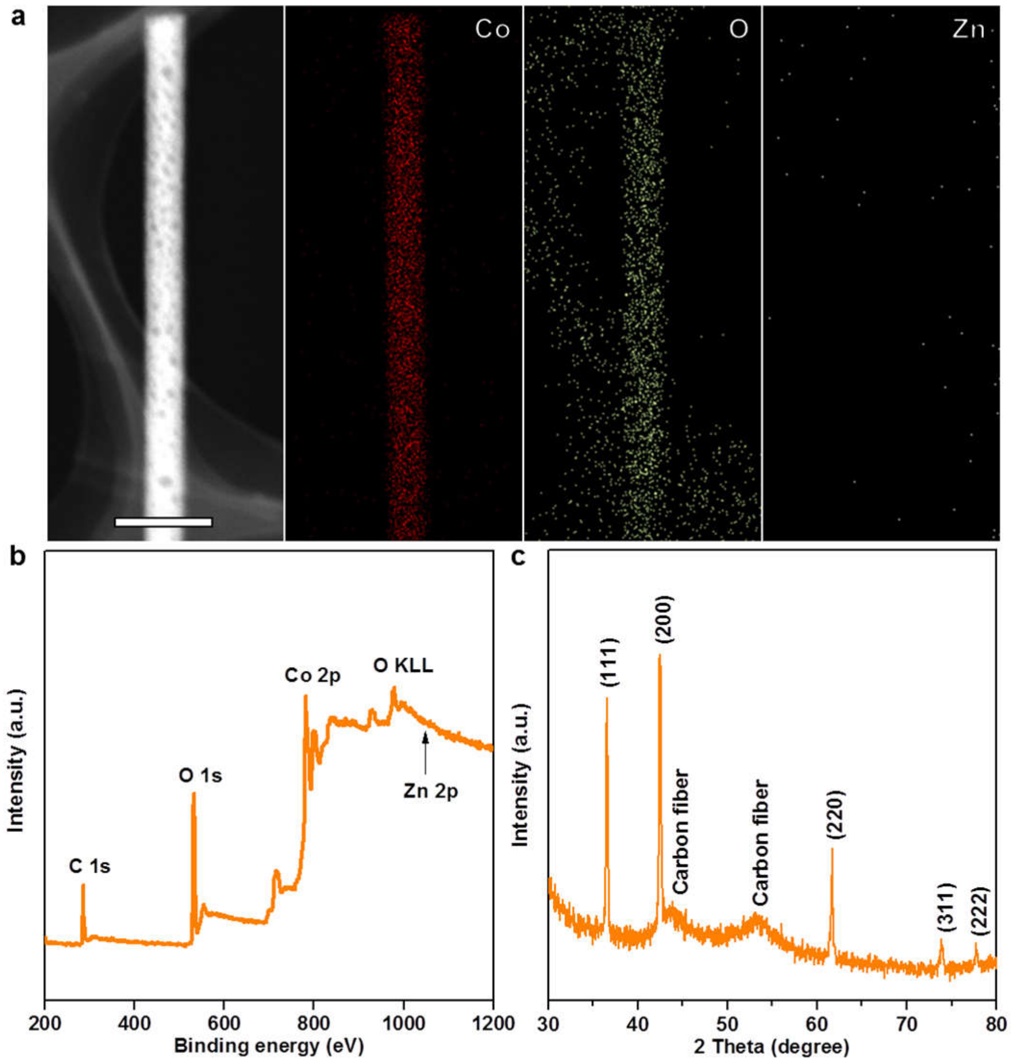

Supplementary Figure 12. Characterization of 3.0 % S-CoO NRs. a, EDS mapping of an individual 3.0 % S-CoO NR. Scale bar, 200 nm. It shows that ZnO has been completely transformed into CoO. **b** and **c**, XPS and XRD patterns of 3.0 % S-CoO NRs, respectively.

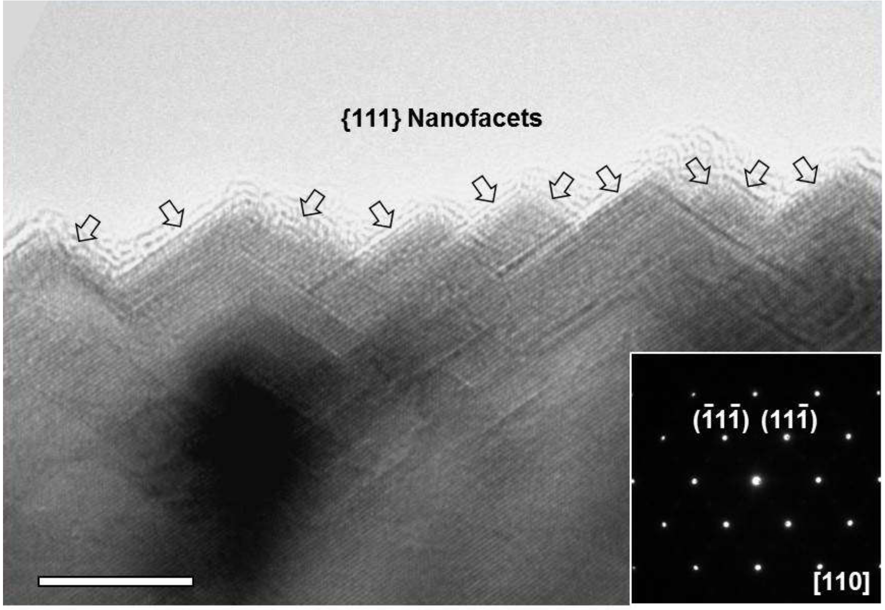

Supplementary Figure 13. High resolution TEM (HRTEM) image of an individual 3.0 % S-CoO NR. The HRTEM image shows that the outermost surface of as-exchanged CoO NR enclosed with {111} nanofacets, with inset presenting the selected area electron diffraction (SAED) pattern in [110] zone axis. Scale bar, 10 nm.

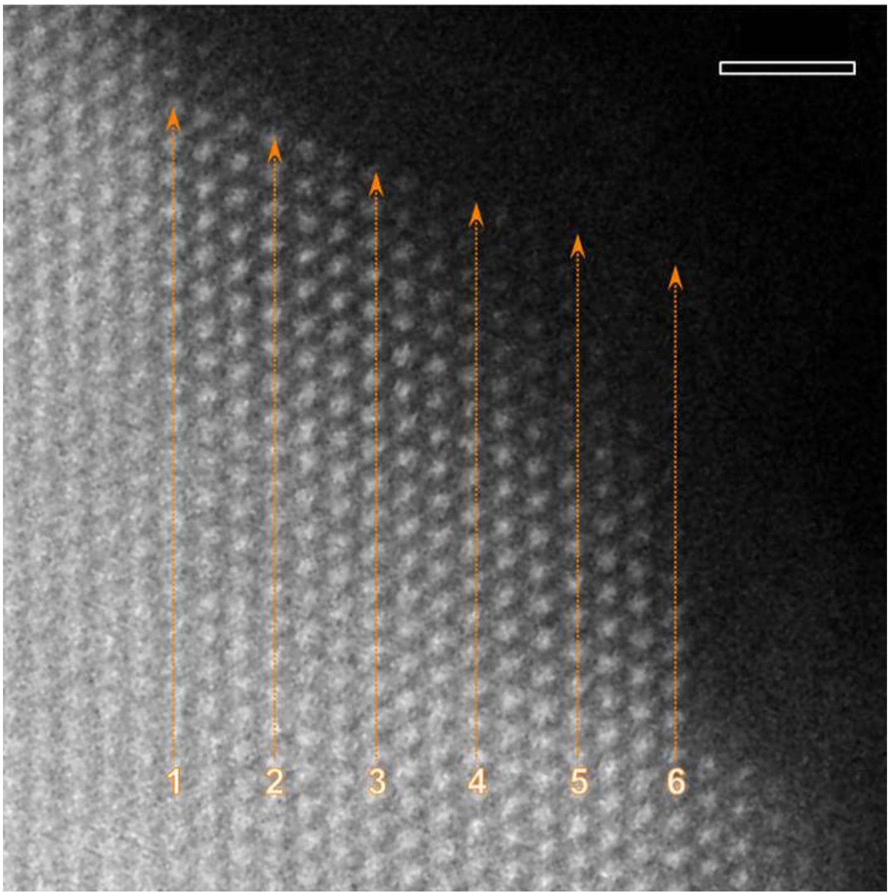

Supplementary Figure 14. High-angle annular dark field-scanning transition electron microscopy (HADDF-STEM) image of an individual nano-sawtooth structure on the outermost surface of a single 3.0 % S-CoO NR. Scale bar, 1 nm. The orange arrows clearly illustrate the curvature of {111} planes progressing from the internal to the external part of the nano-sawtooth structure. The curvature of plane 5 even reaches the scale of an atomic size.

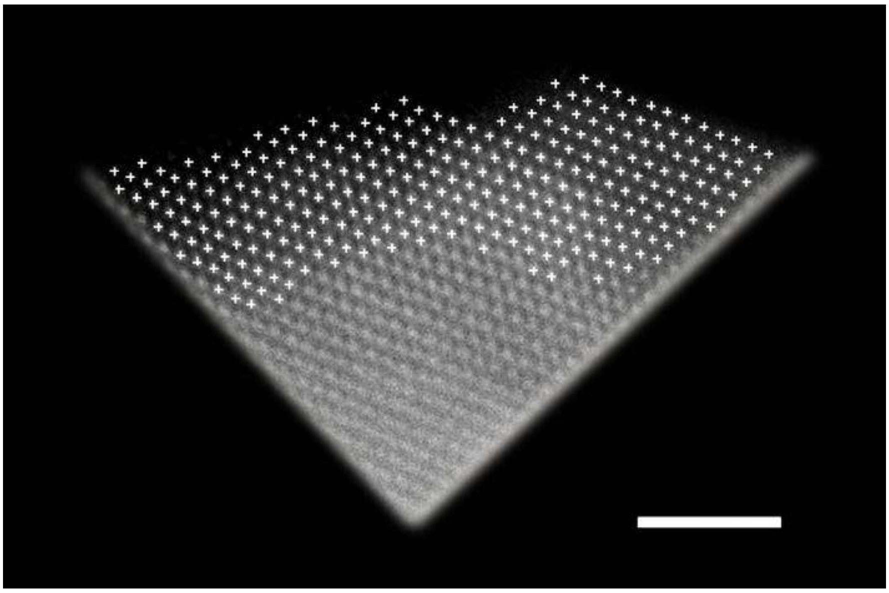

Supplementary Figure 15. Analysis of average strain on the outermost surface of a single 3.0 % S-CoO NR. Atomic columns considered in the calculation are indicated by white crosses. Scale bar, 2 nm.

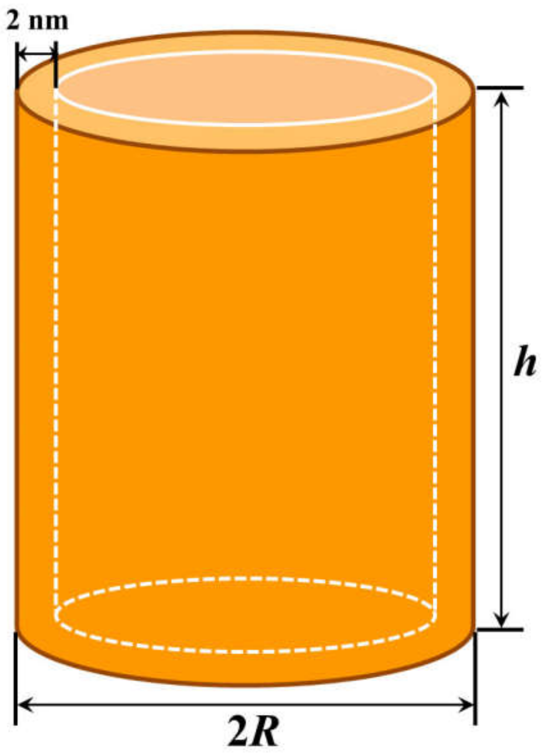

Supplementary Figure 16. Illustration of an individual CoO NR with strained surface. As shown in Fig. 2, HADDF-STEM image provides the strain information of the surface-specific volume (below 2 nm from the outermost surface of CoO NRs), and X-ray diffraction (XRD) analysis gives the average strain information on the whole volume of CoO NRs.

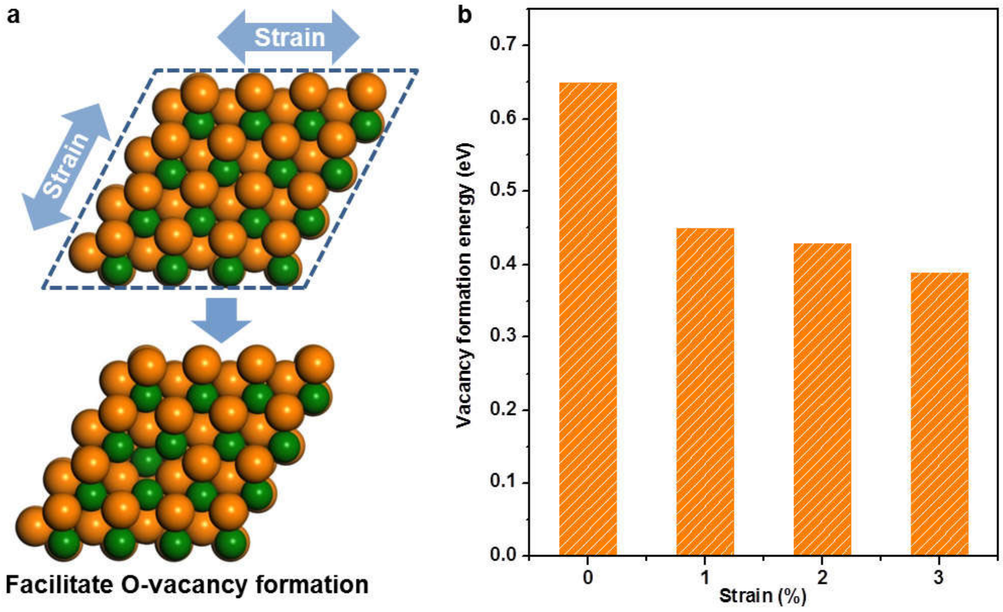

Supplementary Figure 17. Correlation between strain and O-vacancy formation energy on the surface of CoO {111}-O surface. a, Schematic diagram of tuning the O-vacancy formation by strain. **b**, The change in the O-vacancy formation energy obtained for CoO {111}-O surface as a function of tensile strain.

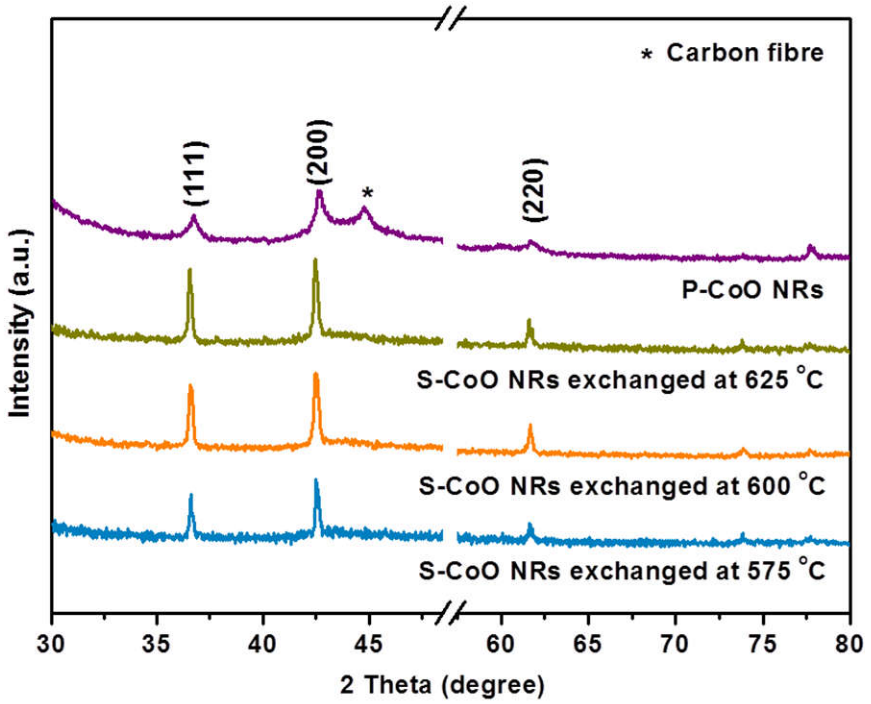

Supplementary Figure 18. XRD spectra of S-CoO NRs prepared by cation exchange method at various temperatures and P-CoO NRs.

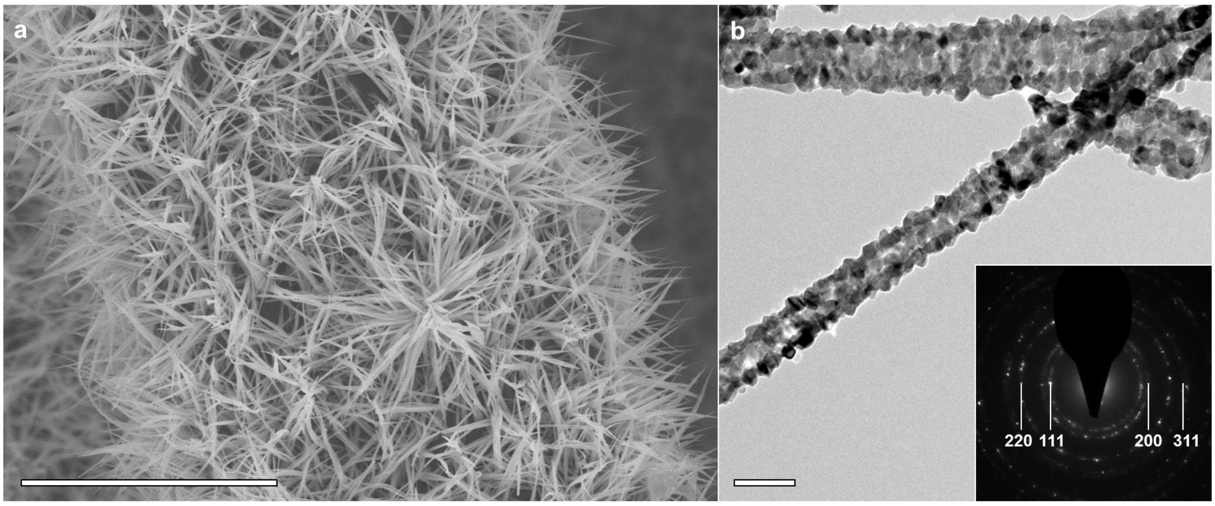

Supplementary Figure 19. Characterization of P-CoO NRs fabricated on CFP. a and **b**, SEM and TEM images of P-CoO NRs, respectively. The inset in **b** denotes the SAED pattern of P-CoO NRs, with polycrystalline rings corresponding to (111), (200), (220) and (311) rings of the face-centered cubic phase of CoO. Scale bars in **a** and **b**, 5 µm and 100 nm, respectively.

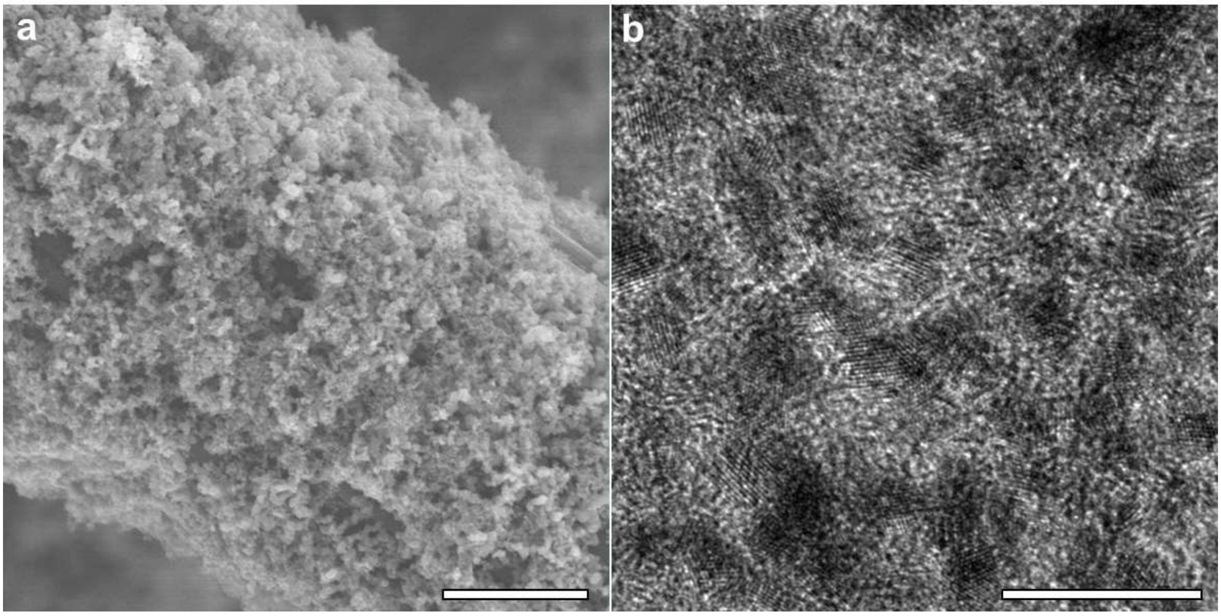

Supplementary Figure 20. Characterization of Pt/C catalysts supported on CFP substrate. a, Low magnification SEM image. Scale bar, 2 μm. **b**, HRTEM image. Scale bar, 10 nm.

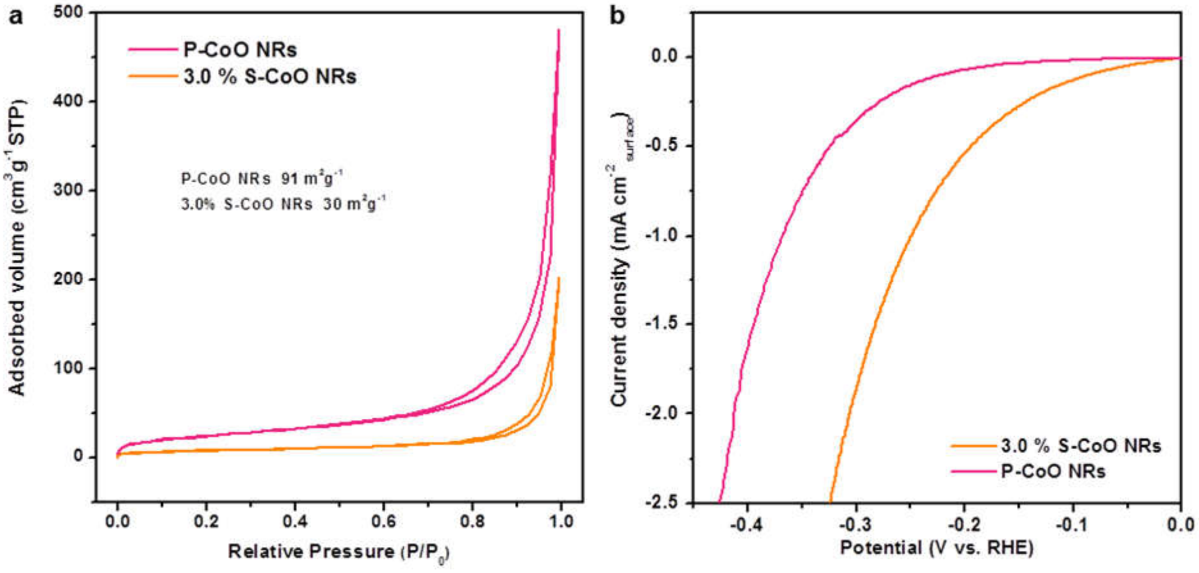

Supplementary Figure 21. Comparison of the HER activity for 3.0 % S-CoO NRs and P-CoO NRs. a, Nitrogen adsorption-desorption isotherms and the corresponding Brunauer-Emmett-Teller (BET) surface area values obtained for 3.0 % S-CoO NRs and P-CoO NRs. **b**, Linear sweep voltammetry (LSV) of 3.0 % S-CoO NRs and P-CoO NRs in 1 M KOH with the current density normalized with respect to the surface area. As shown in **b**, the HER activity of 3.0 % S-CoO NRs is overwhelmingly superior in comparison to that of P-CoO NRs.

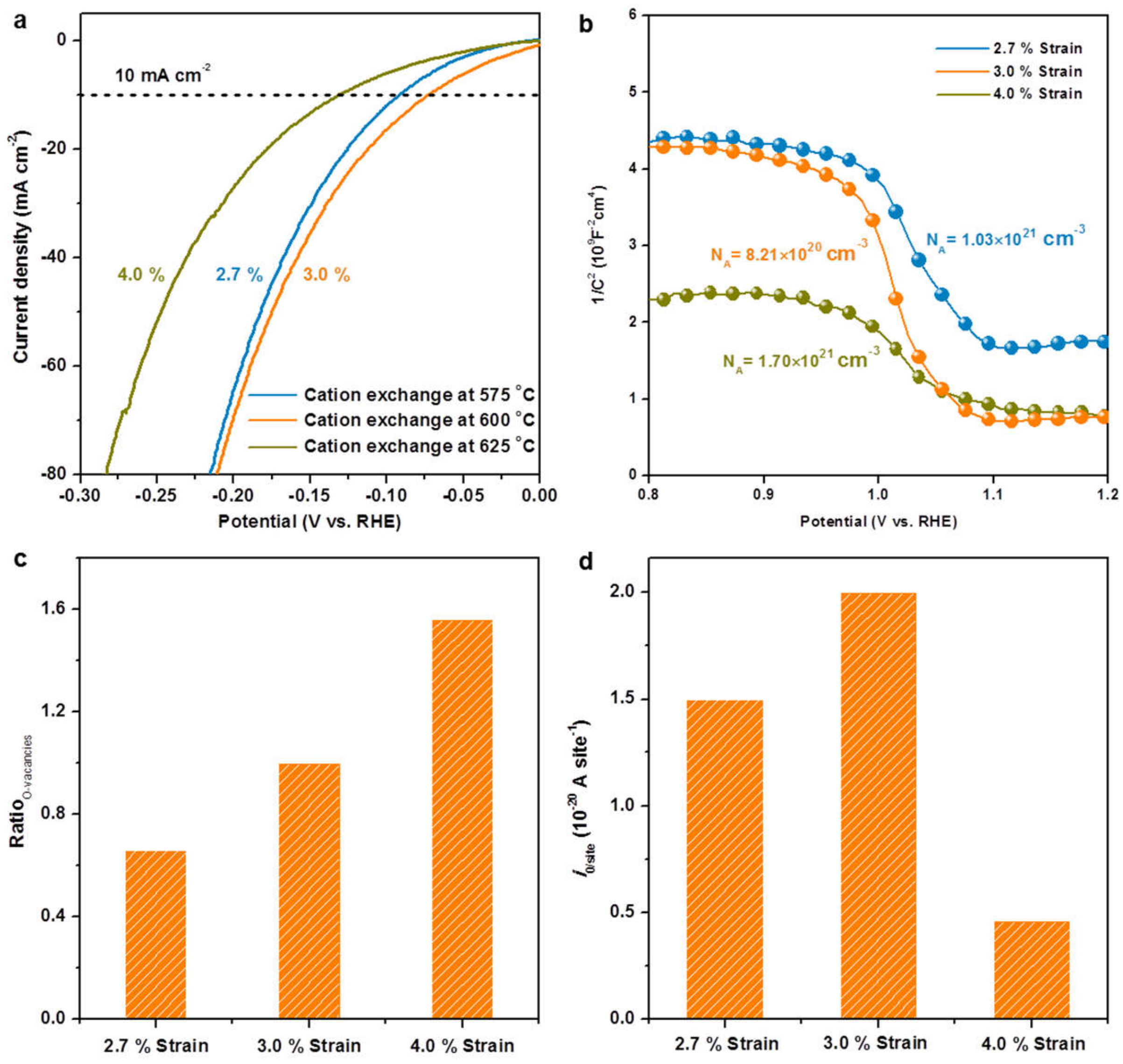

Supplementary Figure 22. HER activity of S-CoO NRs with different tensile strain. a, LSV in 1 M KOH. **b**, Mott-Schottky (M-S) plots recorded with the frequency of 1000 Hz. The carrier concentrations calculated on the basis of the M-S plots[3] are provided; the carrier concentration of 3.0 % S-CoO NRs is not larger than that of the two other samples. **c**, The ratios of O-vacancies present on the surface of strained CoO NRs. The number of O-vacancies on each sample was normalized in reference to that on the surface of 3.0 % S-CoO NRs. **d**, Exchange current per active site, $i_{0/site}$ (Normalization of the exchange current with respect to the total O-vacancies on the S-CoO NRs, Supplementary Table 2).

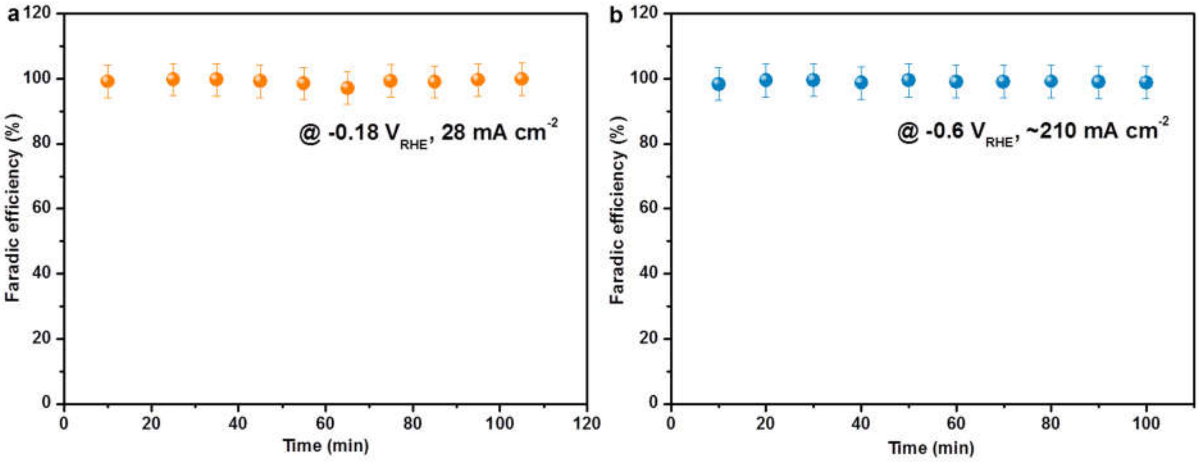

Supplementary Figure 23. Faradaic efficiency of 3.0 % S-CoO NRs from gas chromatography measurement of evolved H_2 to within our available ±5 % experimental error. It shows that 3.0 % S-CoO NRs give ~100% Faradaic yield with both relative (**a**) low (28 mA cm^{-2}) and (**b**) high (~210 mA cm^{-2}) HER current, indicating no other side reactions going on during HER.

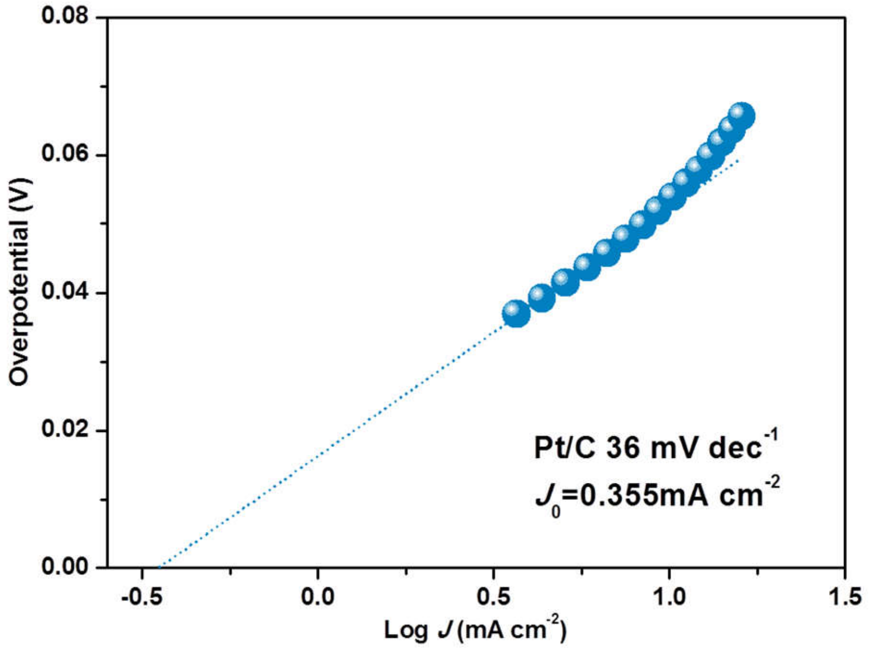

Supplementary Figure 24. Calculation of the exchange current density, J_0, of Pt/C catalysts by the linear fitting of Tafel plot. The calculated J_0 of Pt/C is 3.55×10^{-4} A cm^{-2}, which agrees well with the reported data[4].

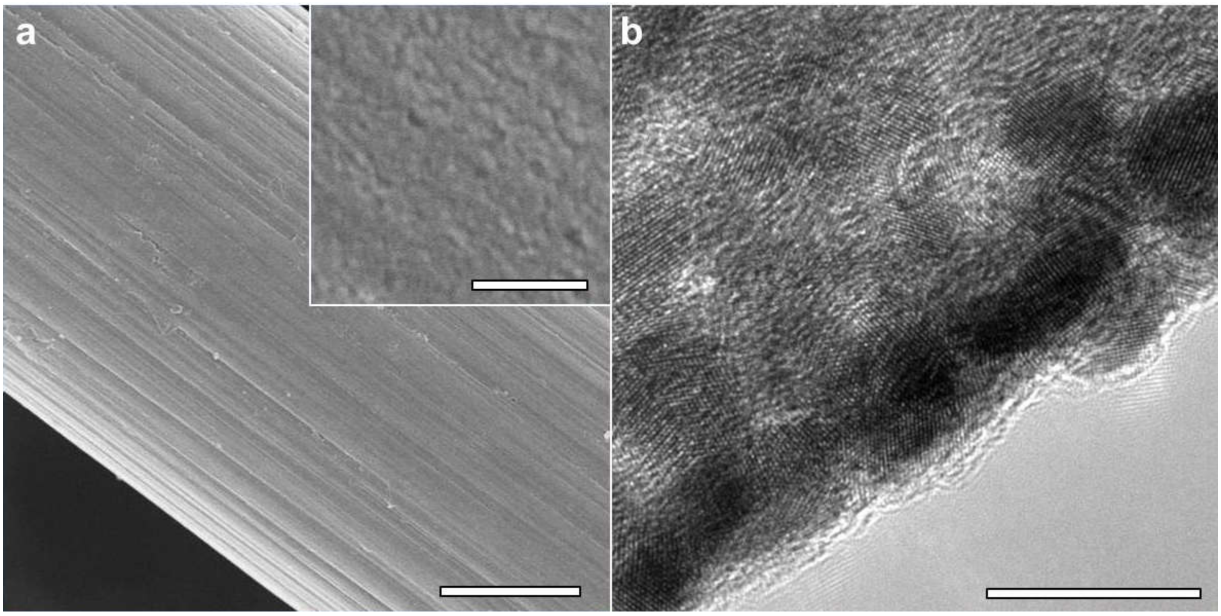

Supplementary Figure 25. Characterization of Pt catalysts supported on CFP substrate. a, Low magnification SEM image. Scale bar, 2 μm. The inset is the corresponding high magnification SEM image. Scale bar, 100 nm. **b**, HRTEM image. Scale bar, 10 nm.

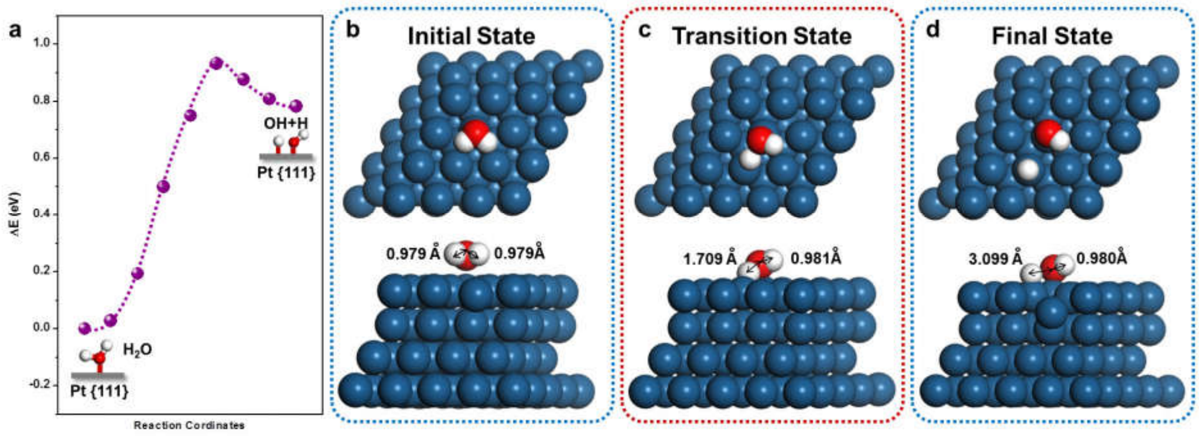

Supplementary Figure 26. Water adsorption and dissociation on Pt {111} surface. a, Water dissociation barrier. **b-d**, Atomic configurations of water dissociation steps. Color codes: white and red spheres denote hydrogen and oxygen atoms in water molecule, respectively, and light blue spheres denote Pt atoms. The calculated water dissociation barrier on Pt (111) is 0.93 eV, which is in good agreement with the recently reported data[5].

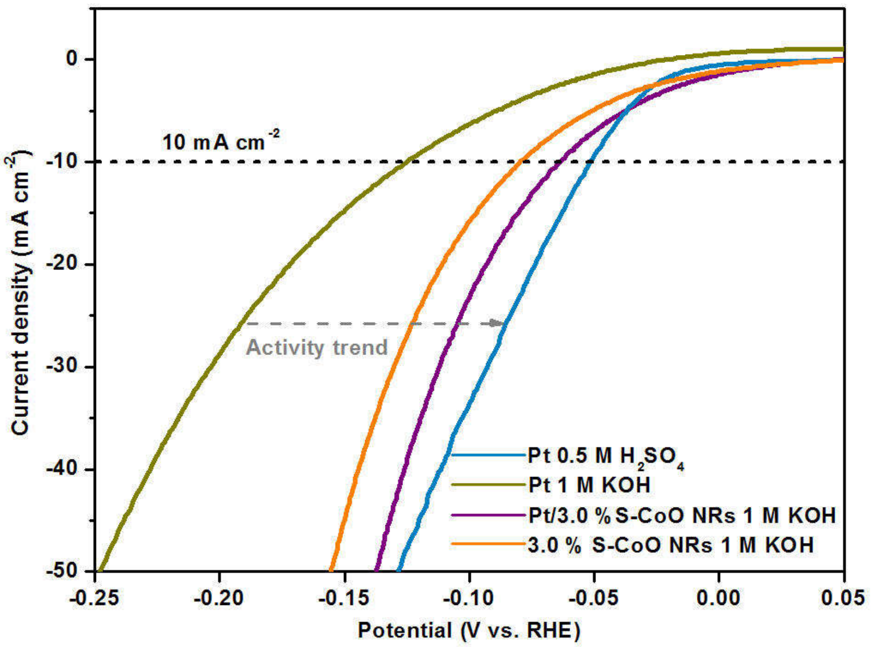

Supplementary Figure 27. Comparison of the HER activities of Pt, 3.0 % S-CoO NRs and Pt/3.0 % S-CoO NRs. Pt nanoparticles (NPs) with the same loading (~0.02 mg cm^{-2}) were directly deposited on the CFP substrate and 3.0 % S-CoO NRs using magnetron sputtering method, followed by thermal treatment to assure good adhesion of Pt NPs on CFP or 3.0 % S-CoO NRs.

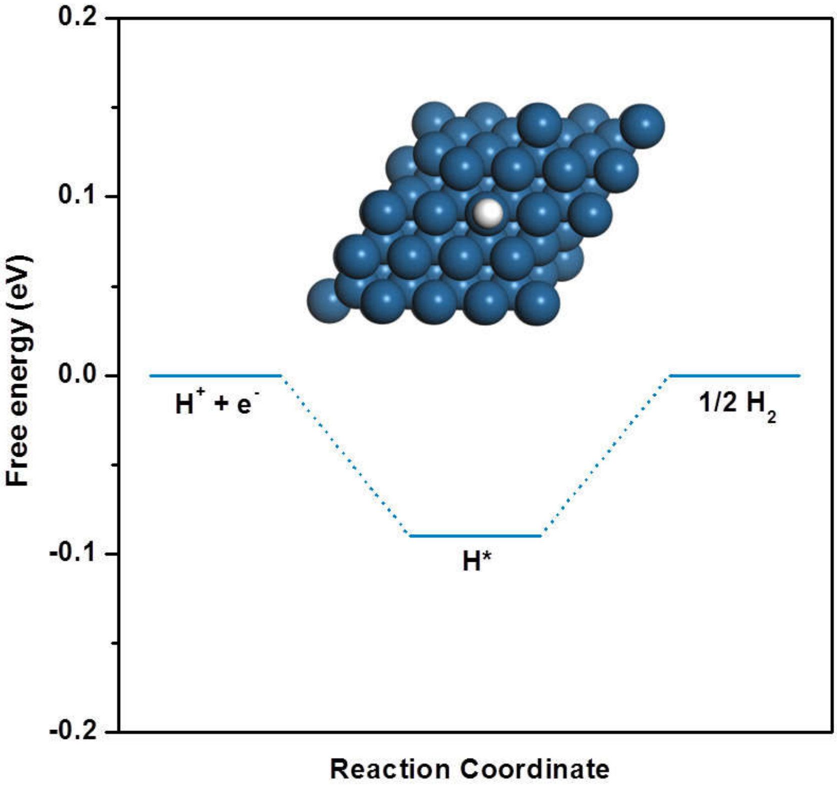

Supplementary Figure 28. Free-energy diagram for HER on the Pt (111) surface. Color codes: light blue and white spheres represent Pt and H atoms, respectively. The value of the calculated hydrogen adsorption free energy, ΔG_{H*}, is identical with the recently reported data[5].

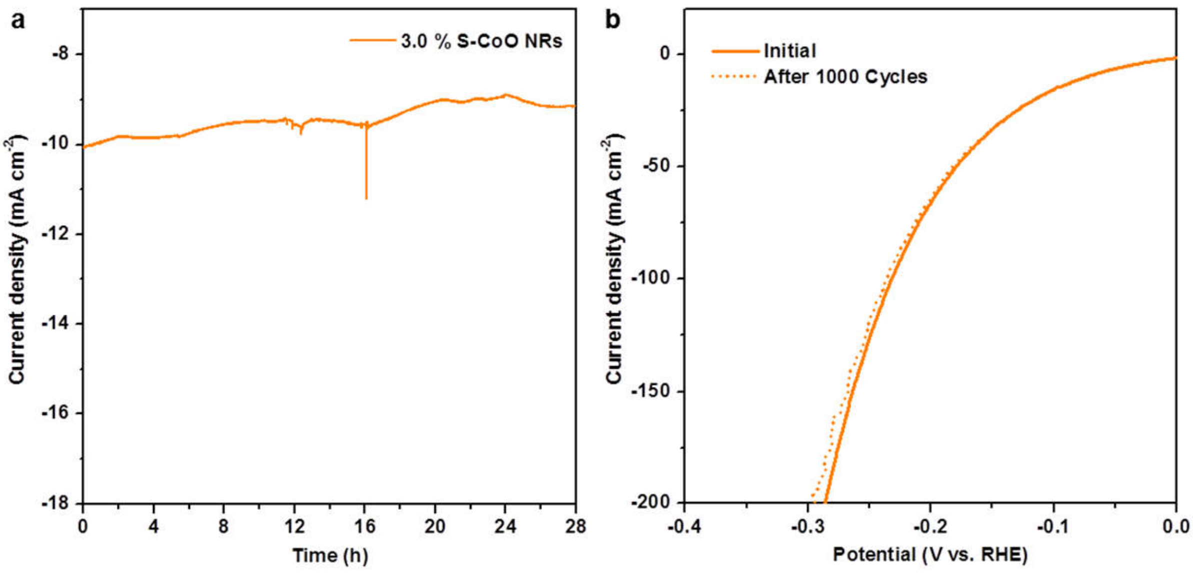

Supplementary Figure 29. Evaluation of the stability of 3.0 % S-CoO NRs. a, Current-time (*i*-*t*) chronoamperometric response of 3.0 % S-CoO NRs at an overpotential of -0.073 V_{RHE}. **b**, Accelerated durability test (ADT). HER polarization curves recorded before and after 1000 cyclic sweeps with scan rate of 100 mV s^{-1} between -0.6-0.2 V_{RHE} in 1 M KOH.

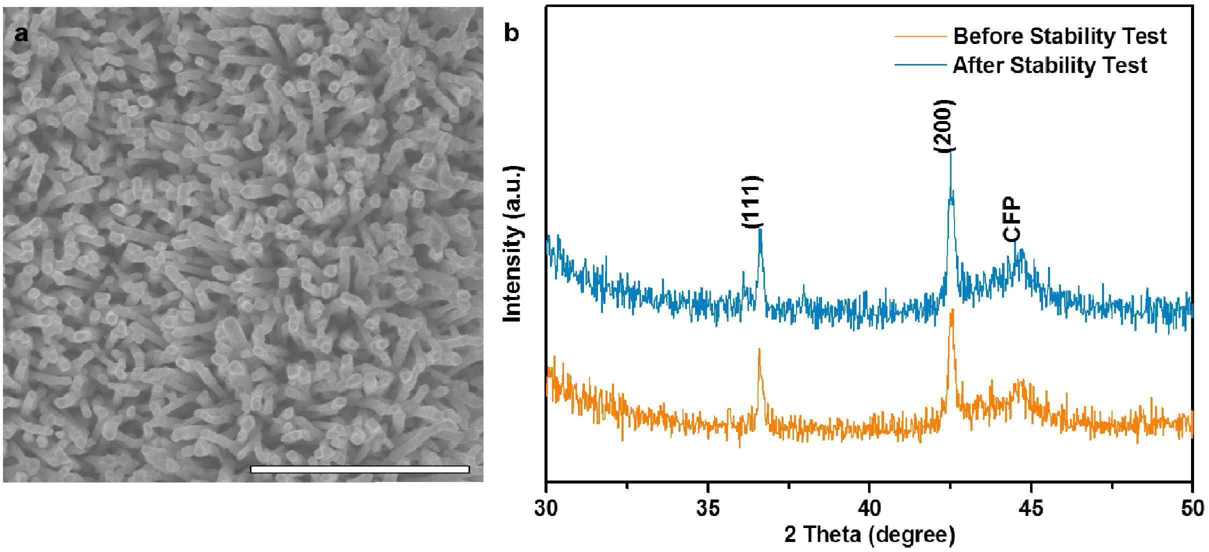

Supplementary Figure 30. Characterization of 3.0 % S-CoO NRs after ADT test. a, SEM image. Scale bar, 2 µm. **b**, XRD spectra.

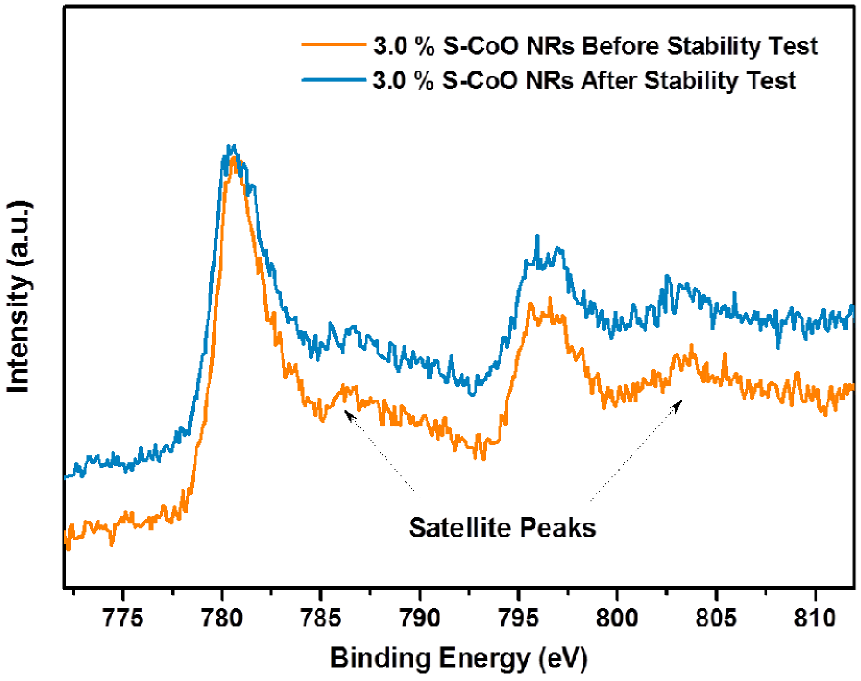

Supplementary Figure 31. XPS Co 2p spectra of 3.0 % S-CoO NRs before and after ADT tests. No obvious intensity decrease in the satellite peaks indicates that NRs after ADT test are mainly CoO. Moreover, no noticeable peak shift was observed on the Co 2p XPS spectrum of 3.0 % S-CoO NRs after stability test in comparison to that of the fresh sample, suggesting the majority of O-vacancies are preserved during the HER.

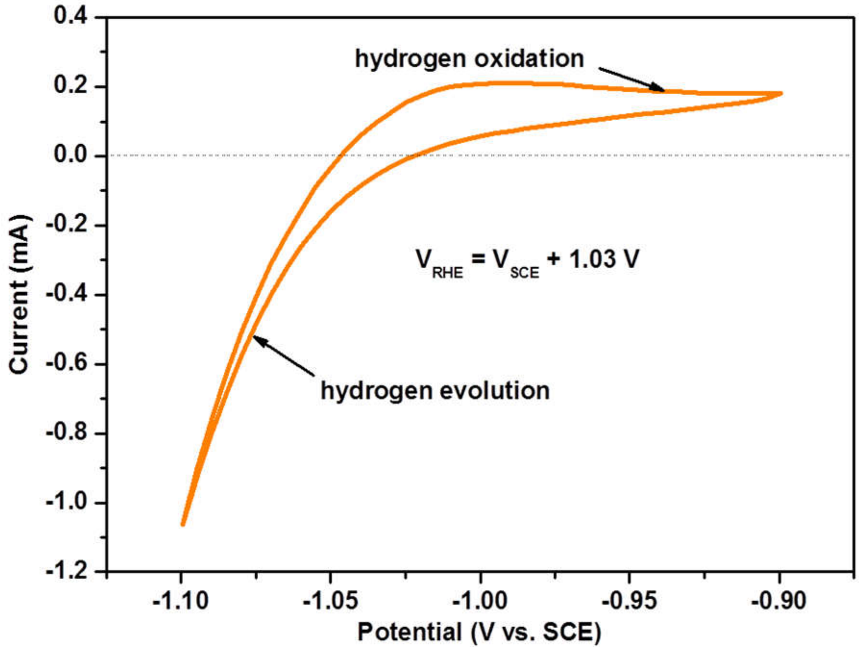

Supplementary Figure 32. Calibration of the reference saturated calomel electrode (SCE). The calibration was performed in hydrogen saturated electrolyte with a Pt sheet as the working electrode. Cyclic voltammetry run at a scan rate of 1 mV s^{-1}, and the average of the two potentials at which the current value was zero was taken as the thermodynamic potential. Therefore, in 1 M KOH, $V_{RHE} = V_{SCE} + 1.03$ V.

Supplementary Tables

Supplementary Table 1. Bader charges of adsorbed H atom, O atom (forming OH bond with adsorbed H atom), total O and CoO atoms in the super cell (Supplementary Fig. 7) based on Bader charge analysis.

	Bader charge (e)			
Strain (%)	Q_H	Q_O	$Q_{Total\ O}$	$Q_{Total\ Co}$
0	0.314	7.503	559.350	493.900
3	0.328	7.445	559.900	493.360

Supplementary Table 2. Structural parameters of S-CoO NRs exchanged at different temperatures and P-CoO NRs.

Sample	Surface tensile strain (%)	Lattice constant[a] (Å)	Composition	Oxygen vacancy concentration[b], δ
S-CoO NRs exchanged at 575 °C	2.7	4.250	$CoO_{0.979}$	0.021
S-CoO NRs exchanged at 600 °C	3.0	4.251	$CoO_{0.968}$	0.032
S-CoO NRs exchanged at 625 °C	4.0	4.254	$CoO_{0.950}$	0.050
P-CoO NRs	-	4.241	-	-

[a]From XRD Rietveld refinement.

[b]Average data from inductively coupled plasma mass spectrometry (ICP-MS) measurements.

Supplementary Table 3. Calculated hydrogen adsorption free energies (ΔG_{H^*}) on the CoO {111}-Ov surface with varied lattice strain. The O-vacancy concentration on this surface is 11.1%.

	Strain (%)				
	0	1.0	2.0	3.0	4.0
ΔG_{H^*} (eV)	-1.22	-0.66	-0.41	-0.10	0.55

Supplementary Table 4. Summary of the recently reported highly active HER catalysts in alkaline and acidic solutions.

Catalyst	Loading (mg cm^{-2})	Electrolyte	Overpotential @ 10 mA cm^{-2} (mV)	Tafel slope (mV dec^{-1})	Reference
**S-CoO NRs**	**0.486**	**1 M KOH**	**73**	**82**	**This work**
**P-CoO NRs**	**0.4**	**1 M KOH**	**208**	**164**	**This work**
**Pt/C**	**0.4**	**1 M KOH**	**53**	**36**	**This work**
**NiO/Ni-CNT**	**0.28**	**1 M KOH**	**80**	**82**	**[6]**
**2-Cylce $NiFeO_x$**	**1.6**	**1 M KOH**	**88**	**150**	**[7]**
**Ni-Mo nanopowder**	**1.0**	**2 M KOH**	**70**	**--**	**[8]**
**CoP**	**2.61**	**1M KOH**	**94**	**42**	**[9]**
**$Ni_{12}P_5$**	**3**	**0.5 M H_2SO_4**	**107**	**--**	**[10]**
**$CoSe_2$-CFP**	**2.8**	**0.5 M H_2SO_4**	**137**	**42**	**[11]**

Supplementary Notes

Supplementary Note 1. Investigation water dissociation on typical CoO surfaces

We performed DFT calculations to gain insight into the energetics and mechanism for the dissociation of water on typical CoO surfaces. On {100} surface, molecular water adsorption is 0.24 eV energetically favourable than the complete dissociative adsorption (Supplementary Fig. 1), indicating that water dissociation is difficult on this surface. In contrast, the latter favours over the former by 1.07 eV on {110} surface (Supplementary Fig. 2). When O-vacancies are introduced on {111}-O surface, water dissociation, resulting in OH healing of the O-vacancy and the remaining H atom adsorbing on the top of a nearest surface O atom (Fig. 1a), is advantageous over molecular adsorption by 0.67 eV (Supplementary Fig. 3). From a catalytic viewpoint, the moderately bounded H-OH groups on {111}-Ov are expected to be more reactive than in the case of {110} facets.

Supplementary Note 2. Analysis of strain on the surface of S-CoO NRs

Geometric phase analysis (GPA) was used to obtain the strain tensor maps of the surface of S-CoO NRs. According to th e GPA method[12], the derivation of the displacement field gives the strain field:

$$\varepsilon_{11} = \partial u_1(R) / \partial R_1 \tag{1}$$

$$\varepsilon_{22} = \partial u_2(R) / \partial R_2 \tag{2}$$

$$\varepsilon_{12} = 1/2(\partial u_1(R) / \partial R_2 + \partial u_2(R) / \partial R_1) \tag{3}$$

where R is a position in the image (Fig. 2b), $u(R)$ is the vectorial representation of the displacement field. The strain determined here can be easily transformed into the strain reference to the lattice parameter (a),

$$\varepsilon_{11} = \frac{R_1^{\text{local}} - R_1^{\text{reference}}}{R_1^{\text{reference}}} = \frac{\sqrt{6}/2(a^{\text{local}} - a^{\text{reference}})}{\sqrt{6}/2 \cdot a^{\text{reference}}} = \frac{a^{\text{local}} - a^{\text{reference}}}{a^{\text{reference}}} \tag{4}$$

where R_1 is the length of unit vector along $\boldsymbol{R_1}$ shown in Fig. 2b, and the value of that inside the S-CoO NRs without strain is selected as $R_1^{\text{reference}}$, correspondingly, the lattice parameter of CoO inside the S-CoO NRs without strain is selected as $a^{\text{reference}}$. Therefore, GPA generates strain maps (Figs. 2c-2e) with color contours directly illustrating the location of the lattice strain. A

comparison of the values of ε_{11}, ε_{22} and ε_{12} reveals that the tensile strain on the surface of S-CoO NRs is biaxial with the average values of ε_{11}=0.033, ε_{12}=0.04 and ε_{22}=0.035 (The considered atoms on the surface of S-CoO NRs are indicated by white crosses in Supplementary Fig. 15).

Supplementary Note 3. Determination the magnitude of tensile strain on the surface of S-CoO NRs

As discussed in the main text, the cation exchange method was employed to fabricate CoO NRs with strained surface. It was found that an increase in the cation exchange temperature results in larger tensile strain on the surface of S-CoO NRs.

The surface-specific strain of S-CoO NRs can be obtained from HADDF-STEM ($\text{Strain}_{\text{surface}}$), while the XRD analysis gives the average information about the whole CoO NRs (Supplementary Fig. 16). The relation between the lattice strain measured by HADDF-STEM and XRD can be correlated using the following equation:

$$\frac{\text{Strain}_{\text{surface}}}{\text{Strain}_{\text{bulk}}} = \frac{V_{\text{bulk}}}{V_{\text{surface}}} \Rightarrow \frac{\text{Strain}_{\text{surface}}}{\text{Strain}_{\text{bulk}}} = \frac{\text{Strain}_{\text{surface}}}{(a_{\text{S-CoO NRs}} \text{-} a_{\text{reference}}) / a_{\text{reference}}} = \frac{\pi R^2 \cdot h}{\pi (R^2 - (R - 2\text{nm})^2) \cdot h} \quad (5)$$

where V_{surface} and V_{bulk} are the surface-region (below 2 nm from the outermost surface of CoO NRs) and whole volume of S-CoO NRs, R and h is the radius and height of the CoO NRs, respectively. The average radius (R) of S-CoO NRs is estimated as 50 nm (Supplementary Fig. 11). Based on the XRD analysis (Supplementary Table 2), the lattice parameter of CoO NRs exchanged at 600 ^{o}C is determined as 4.251 Å, and the lattice constant of P-CoO NRs (4.241 Å) is used as the reference. Hence, the $\text{Strain}_{\text{surface}}$ of S-CoO NRs exchanged at 600 ^{o}C is estimated as 3.0 %, which is comparable to what we have observed by HAADF-STEM (Fig. 2, Supplementary Fig. 15, and Supplementary Note 2). Correspondingly, the magnitude of tensile strain present on the outermost surface of CoO NRs exchanged at 575 and 625 ^{o}C is calculated as 2.7 % and 4.0 %, respectively.

Supplementary Note 4. Analysis of HER activity differences of S-CoO NRs with varied magnitude of tensile strain

The HER activity of S-CoO NRs with 2.7 %, 3.0 % and 4.0 % tensile strain is shown in Supplementary Fig. 22a. As can be seen, 3.0 % S-CoO NRs exhibit the highest activity. The excellent catalytic HER performance of 3.0 % S-CoO NRs may arise from abundant active sites, and/or superior electronic conduction, and/or optimum hydrogen adsorption energy (ΔG_{H^*}). The detailed Mott-Schottky analysis suggests that the electronic conductivity of 3.0 % S-CoO NRs is not better than that of 2.7 % and 4.0 % S-CoO NRs (Supplementary Fig. 22b). As discussed in main text, the O-vacancies present on the surface of S-CoO NRs act as the active sites for HER. The number of O-vacancies on S-CoO NRs with different magnitude of tensile strain was shown in Supplementary Fig. 22c. To decouple the different activity of the S-CoO NRs from contributions due to the strain induced varied hydrogen adsorption energy, ΔG_{H^*}, versus the different quanties of O-vacancies on the surface of S-CoO NRs, the exchange current (i_0) was normalized by the total number of O-vacancies, measured by ICP-MS (Supplementary Table 2), to obtain the normalized i_0 per active site ($i_{0/\mathrm{site}}$). As shown in Supplementary Fig. 22d, 3.0 % S-CoO NRs afford the highest $i_{0/\mathrm{site}}$, consistent with the predicted ΔG_{H^*} that 3.0 % strained {111}-Ov surface has the optimum value of -0.1 eV (Fig. 1b and Supplementary Table 3). Based on these results, it is reasonable to assume that the outstanding HER activity in 3.0 % S-CoO NRs mainly originate from sucessful strain engineering, which fine-tunes the ΔG_{H^*} near to the optimum value that balances hydrogen adsorption and desorption.

Supplementary Note 5. Calibration of Turnover Frequency of 3.0 % S-CoO NRs

As discussed in the theoretical calculations of HER mechanism, O-vacancies on the surface of S-CoO NRs are active sites for HER. It is assumed that all O-vacancies are present on the surface of S-CoO NRs and play role of active sites in the HER process, which can be an underestimation. The total number of O-vacancies on 3.0 % S-CoO NRs was determined to be 3.2 % based on the ICP-MS measurements (Supplementary Table 2). Therefore, the number of O-vacancies, $N_{\text{O-vacancies}}$, on 3.0 % S-CoO NRs was calculated as,

$$N_{\text{O-vacancies}} = \frac{m_{\text{S-CoO NRs}}}{M\mathrm{CoO}_{0.968}} \times N_A \times 0.032 = 1.25 \times 10^{17} \tag{6}$$

where $m_{\text{S-CoO NRs}}$ (0.4806 mg cm^{-2}) is the loading of 3.0 % S-CoO NRs per geometrical area of the electrode, $M_{\text{CoO}_{0.968}}$ is the molar mass of $CoO_{0.968}$, and N_A is Avogadro's constant.

The number of H_2 was calculated using the current density of 3.0 % S-CoO NRs at overpotential of 100 mV via:

$$N_{H_2} = (J\frac{\text{mA}}{\text{cm}^2})\times(\frac{1\text{ C/s}}{1000\text{ mA}})\times(\frac{1\text{ mol e}^-}{96485\text{ C}})\times(\frac{1\text{ mol H}_2}{2\text{ mol e}^-})\times 6.02\times10^{23} = 5.14\times10^{16}\text{ s}^{-1} \quad (7)$$

The turnover frequency (TOF) of 3.0 % S-CoO NRs at overpotential of 100 mV was calculated using the following equation:

$$\text{TOF} = \frac{N_{\text{H}_2}}{N_{\text{O-vacancies}}} = 0.41\text{ s}^{-1} \quad (8)$$

Supplementary Note 6. Comparison of intrinsic alkaline HER activity of 3.0 % S-CoO NRs and Pt/C catalysts

As discussed in the main text, 3.0 % S-CoO NRs exhibit an extremely high exchange current density (J_0) of 2.34×10^{-3} A cm^{-2}, which is significantly larger than that of the-state-of-the-art Pt/C catalysts (3.55×10^{-4} A cm^{-2}, Supplementary Fig. 24) measured under identical conditions. We compare the computational results related to the thermodynamics and kinetics of HER on Pt and CoO surfaces to reveal the origin of the superior activity of 3.0 % S-CoO NRs in alkaline solutions. A Pt slab with (111) plane that represents the Pt/C was constructed (Supplementary Fig. 26). As shown in Supplementary Fig. 28, the hydrogen adsorption on Pt (111) surface is optimal with a free energy (ΔG_{H*}) value of -0.09 eV, which is consistent with the recently reported data[5]. However, for HER in alkaline solutions, the dissociation of water supplies hydrogen and is considered as a key rate determining step[13]. Unfortunately, molecular water adsorption is about 0.78 eV energetically favourable than the complete dissociative adsorption on Pt (111) with an energy barrier of 0.93 eV, indicating that water dissociation is not energetically favourable on this surface (Supplementary Fig. 26). In contrast, the {111}-Ov surface of S-CoO NRs significantly enhances water dissociation over molecular adsorption (Supplementary Fig. 3), suggesting the facile water dissociation on S-CoO NRs.

To verify this conclusion, a comparison of the HER activities of Pt, 3.0 % S-CoO NRs and Pt/3.0 % S-CoO NRs hybrid catalysts was carried out. Pt nanoparticles (NPs) with the same loading (~0.02 mg cm^{-2}) were directly deposited on the CFP substrate (Supplementary Fig. 25)

and 3.0 % S-CoO NRs using magnetron sputtering method, followed by thermal treatment to assure good adhesion of Pt NPs on CFP or S-CoO NRs. The HER polarization curves of 3.0 % S-CoO NRs and Pt/3.0 % S-CoO NRs were recorded in 1 M KOH, while the HER activity of pure Pt NPs was tested both in 1 M KOH and 0.5 M H_2SO_4. As shown in Supplementary Fig. 27, the HER activity of Pt/3.0 % S-CoO NRs is significantly enhanced as compared with that of Pt NPs in alkaline solution and even close to that of Pt NPs in acidic solution, demonstrating that 3.0 % S-CoO NRs must play a promoting role in the dissociation of H_2O and thereby enhances the rate of formation of H* intermediates on the Pt surface. Furthermore, the comparable activity of 3.0 % S-CoO NRs and Pt/3.0 % S-CoO NRs confirms that the hydrogen adsorption kinetics on 3.0 % S-CoO NRs is close to that on Pt surface, which is in good agreement with the predicted ΔG_{H^*} values for 3.0 % S-CoO NRs (-0.1 eV) and Pt (111) surface (-0.09 eV).

Above theoretical and experimental results unambiguously demonstrate the facile water dissociation and favourable hydrogen adsorption energetics on 3.0 % S-CoO NRs, which directly leads to extremely high intrinsic alkaline HER activity relative to state-of-the-art Pt/C catalysts.

Supplementary Methods

Computational methods. Density functional theory plus U (DFT+U) calculations were carried out using projector augmented wave (PAW)[14,15] pseudopotential and the Perdew-Burke-Ernzerhof (PBE) exchange-correlation functional[16], as implemented in the Vienna Ab-initio Simulation Package (VASP) package[17,18]. For a better description of the Co 3*d* electrons, an effective U parameter of 3.7 eV under the approximation introduced by Dudarev *et al.*[19] was applied.

All calculations were carried out using a plane wave kinetic energy cut-off of 400 eV. All structures in the calculations were spin-polarized and relaxed until the convergence tolerance of force on each atom was smaller than 0.05 eV. The energy converge criteria was set to be 10^{-5} eV for self-consistent calculations with a gamma-centre 2x2x1 *k*-mesh. All periodic slabs have a vacuum spacing of at least 15 Å. To model the CoO {100} surface, four Co-O layers (128 atoms) with a supercell size of a=b=12.07 Å, c=21.80 Å, α=β=γ=90º were employed. For {110} surface, four Co-O layers (64 atoms) with a=8.5 3Å, b=12.07 Å, c=20.05 Å, α=β=γ=90º were used. The {111}-O surface model consists of four Co layers and five O layers (160 atoms) with a=b=12.07Å, c=29.78Å, α=β=90º, γ=120º, while in the {111}-Ov surface one surface O atom is removed. For both {111}-O and {111}-Ov surfaces, one H layer was introduced to make the slab to obey the electron counting rule, which is a common method used for polar surfaces[20,21]. In calculations, the two bottom layers (plus the H layer for {111}-O and {111}-Ov) were fixed at their optimized bulk-truncated positions during geometry optimization, whereas the rest of atoms were allowed to fully relax.

The computational unit cell of {111}-Ov under tensile strain is shown in Supplementary Fig. 7. Elongating the lattice parameters of the unit cell increased the elastic strain correspondingly. For this unit cell size, the surface O-vacancy concentration is 11.1% (defined as the number of S-vacancies divided by the total number of O atoms on the pristine surface), which is in line with the experimental data (~12.5 %) estimated based on the Co-$L_{2,3}$ edge XANES spectra (Fig. 3c).

Hydrogen adsorption free energy calculation. The hydrogen adsorption free energy, ΔG_{H*}, can be computed using the following equation[22]:

$$\Delta G_{\mathrm{H*}} = \Delta E_{\mathrm{H*}} + \Delta E_{\mathrm{ZPE}} - T\Delta S \quad (9)$$

where ΔE_{H^*}, ΔE_{ZPE} and ΔS are the binding energy, zero point energy change and entropy change of H* adsorption, respectively. In this work, the values of ΔE_{ZPE} and ΔS on the specific CoO surface were determined by vibrational frequency calculation. Note that the exploration of active sites on the specific surface was conducted and chosen according to the most energetically stable adsorption site (Supplementary Figs. 4-6).

Water dissociation barrier calculation. After identification of the initial and final states for water dissociation step on the Pt (111) surface, the energy barrier was located via searching for transition states by climbing image nudged-elastic band (CI-NEB) method[23] implemented in VASP. The transition states were obtained by relaxing the force below 0.05 eV Å^{-1}. Note that the located transition states were further confirmed by frequency analysis.

Calculation of the O-vacancy formation energy. The O-vacancy formation energy $E_{\mathrm{O_V}}$ was calculated using the following equation[24]:

$$E_{\mathrm{O_V}} = E_{\mathrm{Surf\text{-}O_V}} + \frac{1}{2}E_{\mathrm{O_2}} - E_{\mathrm{Surf}} \tag{10}$$

where $E_{\mathrm{Surf\text{-}O_V}}$ is the energy of a surface with an oxygen vacancy, E_{Surf} is the energy of a surface without oxygen vacancies and $E_{\mathrm{O_2}}$ is the energy of a gas phase O_2 molecule.

Structural characterization. XRD characterization was carried out using a Bruker D8 advance XRD. The loading of as-obtained catalysts on CFP was determined using ICP-MS (Perkin-Elmer, NexION 300Q). BET surface area was determined from nitrogen adsorption data measured at 77 K on an ASAP 2020 physisorption analyser (Micromeritics, Inc., USA). For the determination of O-vacancy concentration (δ) on as-exchanged CoO NRs, after the cation exchange process, the CoO NRs were immediately taken out of the tube furnace and the total mass of CoO NRs (m_{CoO}) were accurately measured by the analytical balance. Then, the resulting CoO NRs were totally dissolved in HNO_3, and the mass of Co^{2+} ions ($m_{\mathrm{Co^{2+}}}$) was determined by ICP-MS. The O-vacancy concentration (δ) can be estimated by the following equation,

$$\delta = 1 - \frac{(m_{\mathrm{CoO}} - m_{\mathrm{Co^{2+}}}) / M_{\mathrm{O}}}{m_{\mathrm{Co^{2+}}} / M_{\mathrm{Co}}} \tag{11}$$

where M_{Co} and M_{O} are the molecular weights of Co and O, respectively.

Electrochemical characterization. The Faradaic yield was calculated from the total charge Q(C) passed through the cell and the total amount of hydrogen produced n_{H_2} (mol). The total amount of hydrogen produced was measured using gas chromatography (Agilent 7890B). Assuming that two electrons are used to produce one H_2 molecule, the Faradaic efficiency can be calculated as follows:

$$\eta = \frac{2F \times n_{H_2}}{Q} \quad (12)$$

where F is the Faraday constant.

Supplementary References

1. Pratt, A. *et al.* Enhanced oxidation of nanoparticles through strain-mediated ionic transport. *Nat. Mater.* **13**, 26-30 (2014).
2. Justo, Y. *et al.* Less Is More. Cation exchange and the chemistry of the nanocrystal surface. *ACS Nano* **8**, 7948-7957 (2014).
3. Gleria, M. & Memming, R. J. Charge transfer processes at large band gap semiconductor electrodes: reactions at SiC-electrodes. *Electroanal. Chem. Interfacial* **65**, 163-175 (1975).
4. Zheng, Y. *et al.* High electrocatalytic hydrogen evolution activity of an anomalous ruthenium catalyst. *J. Am. Chem. Soc.* **138**, 16174-16181 (2016).
5. Wang, P. *et al.* Precise tuning in platinum-nickel/nickel sulfide interface nanowires for synergistic hydrogen evolution catalysis. *Nat. Commun.* **8**, 14580 (2017).
6. Gong, M. *et al.* Nanoscale nickel oxide/nickel heterostructures for active hydrogen evolution electrocatalysis. *Nat. Commun.* **5**, 4695 (2014).
7. Wang, H. *et al.* Bifunctional non-noble metal oxide nanoparticle electrocatalysts through lithium-induced conversion for overall water splitting. *Nat. Commun.* **6**, 7261-7261 (2015).

8. McKone, J. R., Sadtler, B. F., Werlang, C. A., Lewis, N. S. & Gray, H. B. Ni-Mo nanopowders for efficient electrochemical hydrogen evolution. *ACS Catal.* **3**, 166-169 (2013).

9. Jiang, N., You, B., Sheng, M. & Sun, Y. Electrodeposited cobalt-phosphorous-derived films as competent bifunctional catalysts for overall water splitting. *Angew. Chem. Int. Ed.* **54**, 6251-6254 (2015).

10. Huang, Z. *et al.* $Ni_{12}P_5$ nanoparticles as an efficient catalyst for hydrogen generation via electrolysis and photoelectrolysis. *ACS Nano* **8**, 8121-8129 (2014).

11. Kong, D., Wang, H., Lu, Z. & Cui, Y. $CoSe_2$ nanoparticles grown on carbon fiber paper: An efficient and stable electrocatalyst for hydrogen evolution reaction. *J. Am. Chem. Soc.* **136**, 4897-4900 (2014).

12. Galindo, P. L. *et al.* The Peak Pairs algorithm for strain mapping from HRTEM images. *Ultramicroscopy* **107**, 1186-1193 (2007).

13. Subbaraman, R. *et al.* Enhancing hydrogen evolution activity in water splitting by tailoring Li^+-$Ni(OH)_2$-Pt Interfaces. *Science* **334**, 1256-1260 (2011).

14. Kresse, G. & Joubert, D. From ultrasoft pseudopotentials to the projector augmented-wave method. *Phys. Rev. B* **59**, 1758-1775 (1999).

15. Blochl, P. E. Projector augmented-wave method. *Phys. Rev. B* **50**, 17953-17979 (1994).

16. Perdew, J. P., Burke, K. & Ernzerhof, M. Generalized gradient approximation made simple. *Phys. Rev. Lett.* **77**, 3865-3868 (1996).

17. Kresse, G. & Hafner, J. Abinitio molecular-dynamics for liguid-metals. *Phys. Rev. B* **47**, 558-561 (1993).

18. Kresse, G. & Furthmuller, J. Efficiency of ab-initio total energy calculations for metals and semiconductors using a plane-wave basis set. *Comput. Mater. Sci.* **6**, 15-50 (1996).

19. Dudarev, S. L., Botton, G. A., Savrasov, S. Y., Humphreys, C. J. & Sutton, A. P. Electron-energy-loss spectra and the structural stability of nickel oxide: An LSDA+U study. *Phys. Rev. B* **57**, 1505-1509 (1998).

20. Li, F., Feng, Y., Hu, Z., Xu J. & Zhang, L. Substrate engineering in stabilizing epitaxial MgO (111) polar ultrathin films: first-principles calculations. *J. Phys.: Condens. Matter* **26**, 315014 (2014).

21. Wang, Z., Pan, N., Li Z. & Yang, J. A first-principles study of ZnO polar surface growth: Adsorption of Zn_xO_y clusters. *J. Chem. Phys.* **139**, 124704 (2013).

22. Norskov, J. K. *et al.* Trends in the exchange current for hydrogen evolution. *J. Electrochem. Soc.* **152**, J23-J26 (2005).

23. Henkelman, G., Uberuaga, B. P. & Jonsson, H. A climbing image nudged elastic band method for finding saddle points and minimum energy paths. *J. Chem. Phys.* **113**, 9901-9904 (2000).

24. Kushima, A., Yip, S. & Yildiz, B. Competing strain effects in reactivity of $LaCoO_3$ with oxygen. *Phys. Rev. B* **82**, 115435 (2010).
